# Linking Human Destruction of Nature to COVID-19 Increases Support for Wildlife Conservation Policies

**DOI:** 10.1007/s10640-020-00444-x

**Published:** 2020-07-11

**Authors:** Ganga Shreedhar, Susana Mourato

**Affiliations:** 1 Department of Psychological and Behavioural Sciences and the Grantham Research Institute for Climate change and the Environment, The London School of Economics and Political Science, London, UK; 2 Department of Geography and Environment and the Grantham Research Institute for Climate change and the Environment, The London School of Economics and Political Science, London, UK

**Keywords:** Narratives, Communication, Conservation, Wildlife, Extinction, Conservation policy, Environmental policy, Prosocial behaviour, Experiment, COVID-19, D62, D64, D83, Q20, Q28, C99

## Abstract

This paper investigates if narratives varying the cause of the COVID-19 pandemic affects pro-wildlife conservation outcomes. In a pre-registered online experiment (N = 1081), we randomly allocated subjects to either a control group or to one of three narrative treatment groups, each presenting a different likely cause of the COVID-19 outbreak: an animal cause; an animal and human cause (AHC); and an animal, human or lab cause. We found that the AHC narrative elicited significantly greater pro-conservation policy support, especially for bans in the commercial trade of wildlife, when compared to the control group. Possible mechanisms driving this effect are that AHC narratives were less familiar, elicited higher mental and emotional engagement, and induced feelings that firms and governments are responsible for mitigating wildlife extinction.

## Introduction

This paper investigates if narratives varying the likely cause of the COVID-19 pandemic influence people’s support for pro-wildlife conservation policies, as well as pro-wildlife behaviours and behavioural intentions. Understanding the cause of the outbreak is important for choosing what we should do to contain it, and to mitigate the risk of future ones. Yet where the coronavirus originated from still remains a mystery. Much uncertainty characterises debates about the origin of the coronavirus, evident from the various and sometimes conflicting narratives concurrently circulating online in news and social media.

Three popular narratives proposing different causal explanations for the coronavirus outbreak are particularly pertinent for people’s engagement with wildlife and environmental conservation. The first narrative is that the SARS-CoV-2 virus causing the COVID-19 outbreak originated in animals, and may have jumped to humans via intermediary animal hosts in a market in Wuhan which sold wildlife (Animal-Cause (AC) narrative). This is the proximate cause of the pandemic. This causal explanation is based on a growing scientific consensus that the virus is most likely zoonotic, and that the pandemic is an instance of a zoonotic spillover i.e. where animal pathogens are transmitted to humans, similar to past infectious epidemics like Swine Flu and Ebola (Andersen et al. 2020; Mallapaty 2020; Cyranoski 2020).

The second narrative takes this reasoning further by suggesting that the ongoing human destruction of nature, via the depletion of wild animals and their habitats, escalates the risks of such zoonotic spillovers since it increases human-animal interactions (Animal + Human-Cause (AHC) narrative). This is the distal cause of the pandemic. It is compatible with the first narrative and frames the increased risk of zoonotic pandemics as an unintended consequence of anthropogenic mass animal extinction and climate change. It has been put forward by leading biologists like Jane Goodall (Thompson 2020) and environmental policymakers like the UN’s environment chief Inger Andersen (Carrington 2020a).

The third narrative, which is often simultaneously shared alongside the other two, suggests that the virus came from a biosecurity lab in Wuhan studying bat coronaviruses (Animal + Human + Lab-Cause (AHLC) narrative). It provides an alternate proximate cause that has been proposed by some prominent political figures like President Trump and more recently the former UK intelligence head Richard Dearlove (Gardener 2020).The virus could have accidentally escaped from the lab (e.g. through an infected lab worker) and it is possible that scientists at the lab might have tweaked the virus’s genome for research purposes before it escaped. But, unlike the other two narratives, there is no publicly available scientific evidence for this cause at present.^1^ This story locates the pandemic’s cause in the ongoing geopolitical rather than anthropogenic environmental change context. Since it proposes an alternate explanation that diverges from the first two stories, it can be conceptualised as a counter-narrative which can increase uncertainty about the cause when it appears alongside the other stories.

**Fig. 1:**
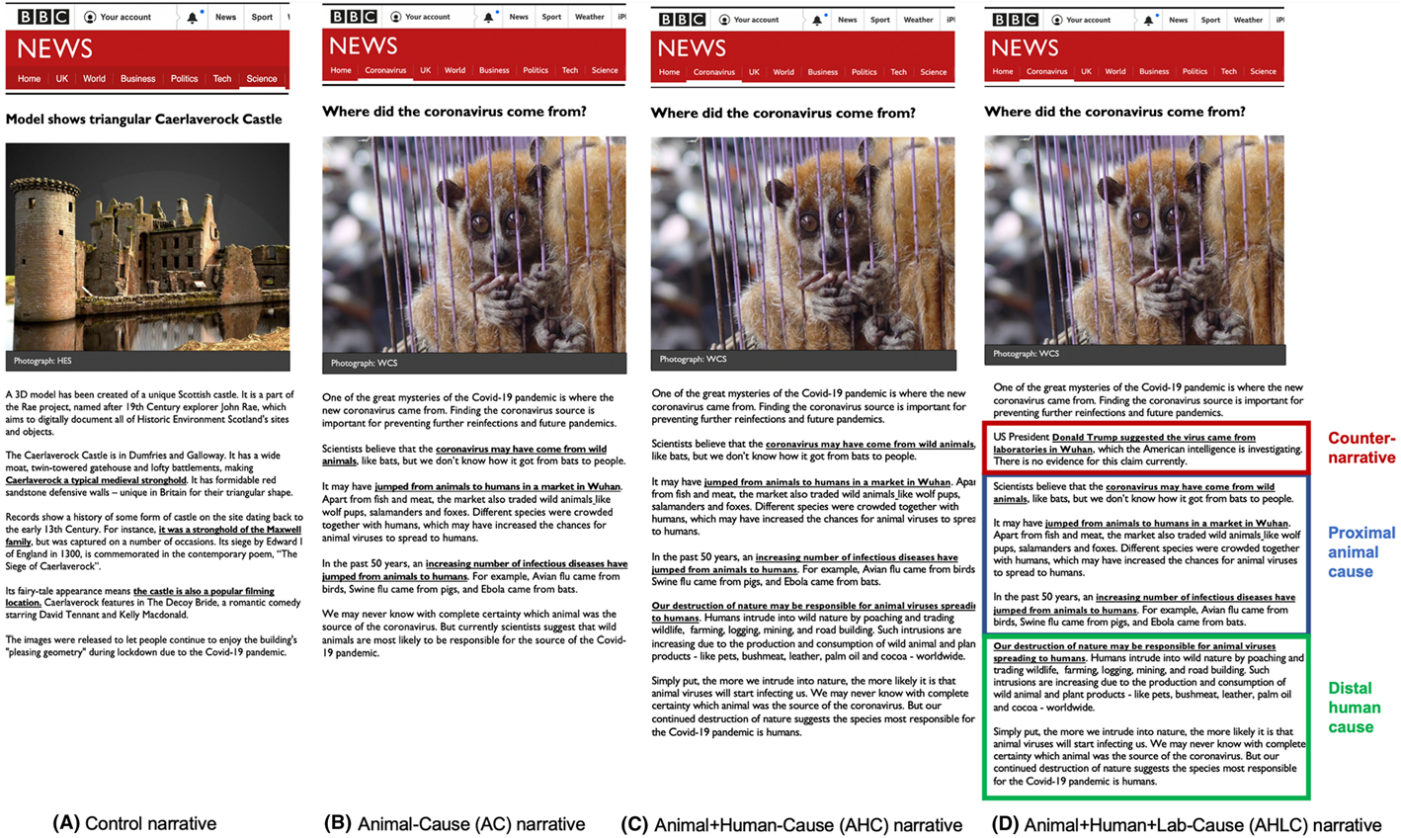
Narrative treatments

To estimate if these types of COVID-19 origin narratives affect pro-conservation outcomes, we ran a pre-registered online experiment in the UK (N = 1081). Subjects were randomly allocated to either a control group (which read a neutral article unrelated to the pandemic) or one of three narrative treatment groups, each presenting a different likely cause of the COVID-19 outbreak: an Animal Cause (AC); an Animal and Human Cause (AHC); and an Animal, Human or Lab Cause (AHLC) (Fig. 1). Then, subjects were asked about a number of pro-wildlife conservation outcomes: donations to nature conservation, in an incentivised charitable giving task, stated intentions to undertake pro-conservation behaviours, and stated support for policies that are pro-wildlife conservation.

To the best of our knowledge, this is the first ever effort to estimate the causal impact of types of COVID-19 origin narratives on pro-wildlife conservation outcomes in a controlled setting. We add a number of new insights to the literature: firstly, providing causal evidence about how types of narratives affects pro-conservation outcomes (e.g. Bénabou et al. (2018)); secondly, estimating the effect of varying the cause of public health challenges (i.e. COVID-19) on support for conservation policies (e.g. Kahneman et al. (1993)); and thirdly, examining the impact of inserting alternate causal explanations into narratives (e.g. van der Linden (2015)).

We found that the AHC narrative elicited significantly greater support for conservation policies, especially for commercial wildlife trade bans, when compared to the control and other treatment groups. Adding the lab story (as in the AHLC group) or removing the human-cause component (as in AC group) attenuated this effect. When we explored possible mechanisms, we found that AHC narratives were less familiar, elicited greater mental and emotional engagement, and induced stronger feelings that firms and governments are responsible for mitigating wildlife extinction. The AHC narrative increased the likelihood of making a donation at the default amount of £10 and over, but neither the AC or any of the other treatment narratives influenced the donation amount or pro-conservation intentions in this setting. Overall, the results suggest that narratives causally linking the human destruction of nature to COVID-19 can increase support for wildlife conservation policies.

Section two reviews the literature on how narratives and causal information shift beliefs, preferences and behaviour. Section three presents the experimental design and materials, and section four the results. Section five concludes with a discussion.

## Related Literature

Narratives, according to Bruner (1991), ‘deal in human or human-like intention and action and the vicissitudes and consequences that mark their course’. They are stories that people tell themselves, and share with others, to make sense of human experience i.e. to organize, explain, justify, predict and sometimes influence its course (Bruner 1991; Chater and Loewenstein 2016; Bénabou et al. 2018). Insights from both economics and psychology suggest narratives—and the causal explanations they provide—can shift support for policies and behaviour to be consistent with the stories told.

In economics, for instance, Bénabou et al. (2018) formalise narratives as rationales or justifications for what one should do (or not) in situations where actions have moral or social implications, such as those involving externalities (also see Shiller 2017; Dolan 2019). They differentiate between ‘responsibilizing’ and ‘absolving’ narratives. The former creates pressure to behave morally, for instance by emphasizing how a person’s actions impact others and moral responsibility. Responsibilizing narratives, therefore, can increase prosocial beliefs, preferences and behaviour. Conversely, absolving narratives employ strategies like blaming the victims, appealing to ‘alternate facts’ and denying responsibility. They can be used to justify antisocial, selfish or short-sighted actions. Both responsibilizing and absolving narratives can operate by changing beliefs about the externality of their choices, or how responsible people feel for (negative) externalities, as long as they are perceived by recipients as containing enough of a ‘grain of truth’. Similarly, Eliaz and Spiegler (2018) consider narratives as causal models of the world which map action variables onto consequences. In their model, ‘lever narratives’ provide causal explanations or levers which trigger specific outcomes and therefore shape policy preferences.

Narrative persuasion and discourse psychology scholars also conceptualise narratives as a type of communication format specifying a cause-effect relationship over time using specific characters (Kreuter et al. 2007; Dahlstrom 2014). They examine how information placed within narratives (e.g. protagonists, plot, emotional valence) elicit responses compared to fact-based or persuasive messages (Green et al. 2004; Kreuter et al. 2007; Moyer-Gusé 2008; Dahlstrom 2010; Braddock and Dillard 2016). Apart from altering beliefs, this strand of research suggests that narratives operate via specific psychological mechanisms such as reducing counter-arguing, changing norms, self-efficacy and outcome expectancies (Moyer-Gusé 2008). A particularly important channel is narrative engagement i.e. narratives work by enabling greater mental, emotional and attentional absorption or ‘transportation’ into the story (Green and Brock 2000; Appel et al. 2015; van Laer et al. 2019). This work, therefore, suggests that exposure to narratives can impact multiple outcomes like beliefs, preferences, intentions and behaviours via several psychological mechanisms.

Yet inconsistent results from empirical research suggest that the effect of narratives is not fully understood. While studies operationalise narratives in numerous ways, most tend to expose subjects to different narratives or formats (e.g. fiction/non-fiction, videos/text) and then measure stated beliefs and behavioural intentions; rely on small convenience samples (e.g. students); and many lack control groups (Winterbottom et al. 2008; Dahlstrom 2010; Greitemeyer 2013; Shen et al. 2015; van der Linden et al. 2015; Braddock and Dillard 2016; Cooper and Nisbet 2016; Moezzi et al. 2017). Recent meta-analyses point to positive associations between narrative exposure and narrative-consistent beliefs, intentions and behaviour (Braddock and Dillard 2016). Shen et al. (2015) highlight that the effectiveness may depend on the issue at hand—they found narrative-format health interventions advocating detection and prevention behaviours led to significant effects, whereas those advocating cessation did not. In the environmental domain, Greitemeyer (2013) found that watching a climate change sceptic film decreased environmental concern relative to a neutral film condition, whereas exposure to a climate change affirming film did not. Similarly, van der Linden (2015) found exposure to anti-climate change conspiracy narratives reduced peoples pro-climate behavioural intentions such as signing petitions, donating or volunteering, compared to a control condition where participants solved a word puzzle. These studies largely focus on the impact of public health or environmental narratives on behaviours in their respective domains. We are not aware of any studies that investigate the effects of narratives connecting health and anthropogenic wildlife extinction, especially in the coronavirus context.

Moreover, although causal information is considered a crucial attribute of all narratives, there is less evidence about how varying the cause affects outcomes. Economists and psychologists have demonstrated that both causal information and the type of cause matters. In environmental valuation studies, for example, people are willing to pay more to address environmental problems caused by humans rather than nature (Kahneman et al. 1993, 1998; Kahneman and Ritov 1994; Brown et al. 2005; Bulte et al. 2005; Böhm and Pfister 2017). Kahneman et al. (1993) termed this the ‘outrage effect’ because they found that people reported human-caused harm as more upsetting than unintentional harm. More recently, Shreedhar and Mourato (2019) found evidence for the outrage effect on wildlife conservation donations. They found that subjects increased donations after exposure to audio-visual narratives causally linking wildlife loss to human causes like poaching and habitat loss, compared to a control group omitting this causal information from the narrative.

Apart from outrage, feelings of responsibility are another mechanism through which the varying type of cause—human versus natural—can influence the willingness to address environmental problems (Walker et al. 1999; Brown et al. 2005; Bulte et al. 2005). In contrast to the outrage effect, the responsibility effect implies that the willingness to pay for human-caused environmental problems is lower than natural ones when people don’t feel responsible for the damages. This is likely in situations when people attribute responsibility to third parties like negligent or polluting firms (Walker et al. 1999; Bulte et al. 2005). Related literature also highlights that ‘felt responsibility’ is an important psychological mechanism that causally links beliefs about human-caused climate change to pro-environmental and climate change mitigation engagement and behaviour (Kaiser and Shimoda 1999; Gifford and Nilsson 2014; Bateman and O’Connor 2016). Bénabou et al. (2018)’s framework also supports the notion that denying or feeling moral responsibility (via absolving or responsibilizing narratives respectively) affects prosocial behaviour.

We contribute to and connect these distinct strands of literature studying the impacts of narratives and causal information by investigating if different causal explanations offered by narratives about a public health challenge (COVID-19) influences pro-wildlife conservation outcomes. Instead of comparing narratives that vary human versus nature-causes of environmental problems, the main focus of past studies, we studied the effect of adding on distal human causes to proximate nature-causes of COVID-19 in the same narrative. In addition, we study if the inclusion of an alternative causal explanation via an absolving narrative (i.e. the lab story) affects outcomes. We motivate our predictions drawing on Bénabou et al. (2018)’s framework of responsibilizing and absolving narratives, and the outrage versus responsibility effect. We conduct exploratory analysis on whether effects are driven by two important psychological mechanisms highlighted in the literature, namely narrative engagement and felt responsibility. We also make a modest contribution by addressing previous methodological limitations in the literature, since we conduct a controlled and pre-registered online experiment using a comparatively larger sample of UK residents, and attempt to elicit both stated behaviour (intentions and policy support) and revealed behaviour (through an incentivised charitable giving task) in the same study.

## Experimental Design and Materials

### Hypotheses

Our main objective is to examine whether different types of narratives about the cause or origin of COVID-19, namely the AC, AHC and AHLC narratives, impact pro-wildlife conservation behaviours, intentions and policy preferences (collectively called pro-conservation outcomes). Since all three narratives raise the issue of COVID-19 originating from wild animals, we expect that they will all elicit higher pro-conservation outcomes when compared to the control group. This is in line with past studies showing how narrative exposure can increase narrative consistent beliefs, preferences, intentions and behaviours (e.g. Braddock and Dillard 2016). So the first hypothesis is that the AC, AHC, and AHLC narratives will increase pro-conservation outcomes when compared to the control narrative.

A second objective is to investigate differences between the AC, AHC, and AHLC narratives. The AHC narrative can be seen as a responsibilizing narrative in Bénabou et al. (2018)’s framework. It creates a moral pressure to increase prosocial pro-conservation outcomes by making the plight of wildlife and nature salient (via trade of wild animals and depletion of their habitats) and identifying human behaviour as the distal cause (as a negative externality of current trade, production and consumption systems). The outrage effect also predicts that human causes may elicit greater pro-conservation outcomes (Kahneman et al. 1993). The AC narrative, on the other hand, does not make the causal link between human-caused mass extinction and COVID-19 explicit. So the second hypothesis is that the AHC narrative will increase pro-conservation outcomes when compared to the AC narrative.

The lab counter-narrative can be seen as an absolving narrative. By adding it to the AHC narrative, as in the AHLC narrative, people might be more likely to justify not engaging in personally effortful (and costly) pro-conservation outcomes. In line with the responsibility effect, people may deny responsibility for COVID-19 by attributing blame to the biosecurity lab which in turn may dampen any increase in pro-conservation outcomes (Walker et al. 1999; Bénabou et al. 2018). That said, since the AHLC treatment group also contains information on the animal and human causes, it is unclear which effect (if any) will dominate. So our third and fourth hypotheses are that the AHC narratives will increase pro-conservation outcomes by the same magnitude as AHLC narrative, and that the AC narrative will increase pro-conservation outcomes by the same magnitude AHLC narrative respectively.

We also undertake further analysis by examining if the impact of different types of narratives is based on past behaviour and beliefs about human-caused mass extinction. In particular, we expect that people who believe more strongly that anthropogenic mass extinction is happening, and who have undertaken pro-conservation behaviours in the past may be more likely to respond by increasing pro-conservation outcomes than those who hold weaker beliefs and have undertaken fewer relevant behaviours in the past. In addition, we also explored if treatment effects depended on past beliefs about the animal cause of the COVID-19 outbreak.

Finally, we undertake exploratory analysis by investigating two possible psychological mechanisms, namely felt responsibility and narrative engagement, which could affect the outcomes considered. Our first exploratory hypothesis is that the AC, AHC and AHLC narratives will increase felt responsibility about wildlife conservation when compared to the control narrative. Our second exploratory hypothesis is that the AC, AHC and AHLC narratives will increase narrative engagement when compared to the control narrative.

### Experimental Procedure

We conducted an online survey experiment. The study was pre-registered on the Open Science Framework.^2^ The survey was programmed on Qualtrics and implemented on 3 June 2020 on the Prolific Academic platform. It was advertised as a study on “Daily life and views during the COVID-19 outbreak”. Participation was open to all UK residents. Ten subjects who previously took part in a pilot and were from the same participant pool were excluded. In addition the survey was restricted to those using laptops and computers to ensure all questions were correctly displayed on the survey interface.

To determine the sample size per treatment group, a power analysis was conducted on G*Power using a non-parametric test of difference in means (Wilcoxon-Mann–Whitney test, two independent groups) (Faul et al. 2007). An effect size of d = 0.25, alpha = 0.05, power = 0.80 was assumed.^3^ This yielded 265 subjects per treatment group. Subjects were excluded if they failed a ‘seriousness check’ in which they could indicate that they did not take part in the survey seriously (Aust et al. 2013).

We recruited 1120 subjects in total to account for possible reductions in the final sample size. A few subjects did not consent to take the survey, dropped off while taking the survey, or failed the seriousness check. The final usable sample consisted of 1081 subjects, or an average of 270 subjects per treatment group. All participants were paid £2 (apart from any earnings from the donation task explained in Sect. 3.4) and the average completion time was around 6 min.

After subjects consented to participate, they answered some introductory socio-demographic questions, followed by questions about the coronavirus and their pro-conservation behaviours and beliefs. Then, each participant was randomly allocated to read one of the four narrative stimuli (explained in Sect. 3.3) after which they could choose to donate money to an environmental charity. They then answered questions about their intentions to undertake more pro-conservation behaviours and their support for different wildlife conservation policies. This was followed by questions about feelings of responsibility and narrative engagement, and the survey concluded with some more exploratory and socio-demographic questions. We attempted to mitigate the possibility of bias arising from order effects in a number of ways (Day et al. 2012): firstly by embedding belief and past behaviour questions amongst other filler questions about people’s daily behaviours and experiences; secondly by randomising the order of these questions; thirdly by randomising the item order within questions (where relevant for e.g. in the multiple-choice questions); and finally by designing the questions to be short and simple in order not to tire the participants.

### Narrative Treatments

Subjects were asked to imagine that they came across an article on their social media feed, and to please read it carefully. They were then randomly assigned to one of the articles containing the narrative treatments and the control.

There were three narrative treatment articles as shown in Fig. 1 (panels B–D). In the AC treatment, the article stated that scientists believe that the coronavirus may have jumped from wild animals to humans in a market in Wuhan. This narrative links the proximate animal cause to the COVID-19 pandemic. The AHC treatment was exactly the same but added causal information explaining that human destruction of nature may be responsible for animal viruses spreading to humans. This narrative links two types of compatible causes, namely the proximate animal and the distal human cause to the COVID-19 pandemic. The AHLC treatment was exactly the same as the AHC but added an alternate causal explanation by stating that President Trump suggested that the coronavirus came from a lab in Wuhan. This narrative presented an alternate explanation as the cause of the COVID-19 pandemic while still making the link with the proximate animal and distal human causes.

The content of the treatment articles was adapted from real online narrative-format articles from the BBC (Briggs 2020), The Guardian (Carrington 2020b; Beaumont 2020), the Daily Mail (Boyd 2020; Pleasance 2020) and the journal Nature (Mallapaty 2020; Cyranoski 2020). As commonly seen in these real articles, our treatment articles also contain one picture placed above the text, for greater realism. Another reason why we chose to place pictures in the narratives is that previous studies pointed out that they enable more effective communication. Pictures can enable us to retain more information via the “picture superiority effect” compared to plain text (Hockley and Bancroft 2011; Schwabish 2014), they can clarify implicit or unclear relations in the text (Eitel and Scheiter 2015) and can facilitate deeper learning via the “multimedia effect” (Mayer 2002). Green and Brock (2002) also suggest narratives with visual images are more engaging and persuasive because pictures enable greater transportation into that narrative.

We followed three criteria in the selection of the final photo: use a real photo of good quality, not explicitly distressing or visceral, and with no people. The picture we selected was a single caged animal to reflect the animal source of the virus. It is also nearly identical to those appearing in BBC articles about the source of the coronavirus (e.g. in Briggs 2020). The animal source is the one common causal explanation across all the treatment groups, so we kept this photo constant across all treatment groups to minimise differences between them, and to balance experimental control with realism. Although we cannot isolate the effect of the picture, its inclusion in the treatment could make the narrative more engaging, memorable and realistic.

Similarly, we designed the text in the treatment articles to be closely aligned with real online narrative-format articles. We used more neutral words in an effort to keep people focused on the arguments presented in the text.^4^ Moreover, the articles were carefully designed to systematically add on different types of causal information in a way that generates greater complexity and uncertainty as we move from the AC to the AHLC treatments. Since we used an add-on design, the addition of new causal explanations increased the word count of each article from 172 in the AC group, to 245 words in the AHC group, to 272 words in the AHLC group. In this way, we attempted to mirror how real COVID-19 origin stories, in online news and social media, often contain multiple causal explanations within the same narrative-format article. While we cannot isolate the effects of word length per se, our approach arguably reflects the multi-layered complex causal connections between environmental and health systems in the COVID-19 context.

Finally, to ensure the treatments were realistic and as similar as possible, we presented them to participants as a snapshot of an online BBC article using the same logo, formatting, font type and colouring. The main message from each paragraph of text in the narrative was underlined in bold in a nearly identical format as online BBC articles, both to help clarify the main message and enable greater comprehension to account for variations in reading ability amongst the participants. The BBC was chosen because recent surveys indicate that it is the most widely used and trusted source of online (and offline) news in the UK across the political spectrum, including both Conservative and Labour voters, and Brexit Leave and Remain voters.

The control narrative was about a Scottish heritage monument. It was adapted from a recent online BBC article to act as a realistic placebo of an article actually in circulation (BBC 2020). It was of a similar length to the AC narrative at 181 words and identically formatted with one picture (Fig. 1, panel A).

### Pro-conservation outcomes

Pro-conservation outcomes included charitable donations (as a revealed behaviour via an incentivised task), behavioural intentions, and support for different wildlife conservation policies.

Donations were elicited from a modified charitable giving task. At the start of the experiment subjects were informed that they had a 1/100 chance to win £20 for successfully completing the survey. After being exposed to the narrative, they were asked how much of that amount, if anything, they wanted to ‘allocate’ to a conservation charity from a pre-selected list of nature conservation and animal charities.^5^ The remaining amount would be sent directly to them with a donation receipt if they won the draw. Subjects used a slider to choose the desired donation amount, that could take any value between £0 and £20, in £1 increments, with the default amount set at £10. We chose this format, including the default, to reflect the practices of real-life charities.^6^

Four self-assessment items were used to measure if subjects were willing to take up relevant pro-conservation behaviours in the future, especially lifestyle and civic actions. These included intentions to avoid eating meat, attend a protest (and other civic actions), engage with a conservation organisation (for instance by donating money or time), and to follow a more sustainable lifestyle (for example by using renewable energy). Response options raged from ‘Definitely not’ (1) to ‘Definitely yes’ (5).

Three self-assessment items measured support for three conservation policies including stricter regulation of the trade and farming of wild animals, a tax on red and processed meat and a ban of most commercial trade in wild animals. Response options ranged from ‘Definitely not’ (1) to ‘Definitely yes’ (5).

### Pro-conservation Covariates and Socio-Demographic Variables

To measure past pro-conservation behaviours, we asked subjects to indicate whether they had engaged with any animal or wildlife organisation, or taken civic action, over the past 2 years. They could indicate if they had undertaken any or some of the following six actions: conservation organisation membership, donating or volunteering (which captured engagement), attended a protest, signed a petition, or written to their MP (which captured civic action). These responses were summed up for each participant to denote how many actions they undertook, such that values ranged from 0 (no actions) to 6 (all six actions). We also asked subjects to describe their diet by selecting one option from a list of the following: omnivore, pescatarian, flexitarian, vegetarian, vegan and restricted diets. We coded this as a dummy variable as 1 (vegetarian or vegan diet) or 0 (other).

To measure past pro-conservation beliefs, we asked subjects to indicate to what extent they agreed with three items that examined anthropogenic mass extinction and environmental change. Statements included ‘Climate change is mostly caused by human activities’, ‘Mass extinction of wild animals is happening’, and ‘Mass extinction of wild animals is mostly caused by human activities’. These items were adapted from Leiserowitz et al. (2010). Response options ranged from ‘Strongly disagree’ (1) to ‘Strongly agree’ (7). These three items were averaged to form a past-proconservation belief score (Cronbach alpha = 0.71).

To measure past animal cause beliefs about the origin of the coronavirus, we asked subjects to indicate to what extent they agreed with one item stating ‘The coronavirus came from animals in a market in Wuhan’. Response options ranged from ‘Strongly disagree’ (1) to ‘Strongly agree’ (7).

In order to check for balance across observables and for further analyses we collected basic sociodemographic data including age, gender, education, self-identified ethnicity, and annual income. We also elicited whether people identified as Leavers or Remainers (or neither) with regards to Brexit.^7^

### Covariates for Exploratory Analysis

Feelings of responsibility for mitigating wildlife extinction was measured by asking subjects how much they agreed with two items: one measuring personal responsibility, and another measuring whether they felt firms and governments have a responsibility to help mitigate wildlife extinction. Response options ranged from ‘Strongly disagree’ (1) to ‘Strongly agree’ (5) and were adapted from (Bateman and O’Connor 2016).

Narrative engagement and familiarity were measured by using three items modified from Appel et al. (2015)’s narrative transportation scale. Subjects were asked to indicate to what extent they felt mentally and emotionally engaged while reading the articles, and whether they were already familiar with the narrative. Responses ranged from ‘Not at all’ (1) to ‘Extremely’ (5).

### Variables for Validity Tests

We assessed if participants recalled information in the narratives by asking them to indicate if they remembered the following: firstly, what the article suggested the cause of the COVID-19 outbreak was (humans due to either the destruction of nature, or a lab, wild animals, none of the above, or don’t remember); and secondly, what other zoonotic diseases were (swine flu, diabetes, none of the above, or don’t remember). Another item asked subjects if they remembered the article source (where options included the BBC, ITV, or CNN). We also asked subjects if they typically used the BBC as a source of online news, and whether they trusted the BBC (where response options were yes, maybe and no). Finally, we measured the amount of time subjects spent on the survey page containing the articles to check if there were any differences in the time spent reading narratives between groups.

We used filler questions throughout and a neutral title (that directed people to their daily life during COVID-19) to address concerns about experimenter demand. To better understand why people chose not to donate, we asked the subset of non-donors to select the reasons for their decision from a list of common responses (e.g. I would rather keep the money, I need to save money, I don’t know the charities etc.).

### Estimation Strategy

To estimate the causal effect of the narratives on donations, we used the Cragg-Hurdle regression model because it allowed us to jointly estimate both the amount donated, as well as the probability of making a donation (by treating £0 as the ‘observed’ lower bound of the donations).^8^ We also explored if narrative exposure increased the likelihood of subjects donating £10 or over (i.e. default donation amount) using a logistic regression. The dependent variable was a categorical variable taking the value 1 if donations were £10 or greater.

For the other pro-conservation outcomes, including pro-wildlife behavioural intentions and policy support, we used ordered logistic regressions since they are ordinal variables.^9^ Wald tests of differences between the treatment categories were used to examine if there was any difference in the treatment effects on policy support. Any heterogeneous effects of past behaviour and beliefs were explored by interacting the treatment dummy with the variable under consideration, and only if it had a significant effect in the main analysis. All statistical models are estimated with robust standard errors.

The main analyses to estimate the average treatment effect is undertaken without covariates for the entire sample of 1081 subjects. Covariates are added in the additional and exploratory analysis for the sample, for those 1008 subjects who chose to disclose their sociodemographic data.

## Results

### Summary Statistics

Table 1 presents summary statistics. Around 69% of subjects identified as being female, 54% were below 34 years of age with only 9% being over 55 years old, almost 90% identified as white, 66% completed an undergraduate degree or attended college, and 65% had an annual income below £50,000. The sample also contained more people identifying as Remainers (65%) than Leavers (25%).

**Table 1:** Summary statistics of socio-demographic attributes and past behaviour and beliefs

Variables	Category	Control	AC	AHC	AHLC	All	*p* value
Age	18–24	22.56	19.63	17.65	19.85	19.91	0.52
	25–34	34.21	34.44	33.09	33.09	33.70	
	35–44	24.44	23.33	27.94	26.84	25.65	
	45–54	9.40	12.22	11.76	11.76	11.30	
	55–64	6.02	6.67	6.99	3.68	5.83	
	65–74	3.01	2.96	2.57	4.78	3.33	
	75–84	0.38	0.74	0.00	0.00	0.28	
Female	Not female*	33.08	28.52	29.41	31.62	30.65	0.65
	Female	66.92	71.48	70.59	68.38	69.35	
Ethnicity	White*	90.23	86.94	90.04	91.14	89.59	0.41
	Not white	9.77	13.06	9.96	8.86	10.41	
Brexit	Leave*	25.56	23.25	25.00	25.37	24.79	0.63
	Remain	66.92	64.94	63.60	64.71	65.03	
	Neither	7.52	11.81	11.40	9.93	10.18	
Education	Primary	0.38	0.00	0.00	0.37	0.19	0.29
	Secondary	9.77	9.26	7.75	7.72	8.62	
	Higher/tech	25.19	27.04	25.83	24.26	25.58	
	UG/Coll.	50.00	45.93	42.07	51.47	47.36	
	> = PG	14.66	17.78	24.35	16.18	18.26	
Income	Under £10 k	4.42	4.90	2.72	5.36	4.35	0.69
	£10–24 k	23.29	21.22	21.01	21.07	21.64	
	£25–49 k	37.75	39.59	38.13	38.70	38.54	
	£50–99 k	29.32	27.76	33.07	29.89	30.04	
	£100–150 k	4.42	4.49	3.11	3.83	3.95	
	> = £150 k	0.80	2.04	1.95	1.15	1.48	
Veg + Vegan	Yes	7.89	9.59	11.40	11.40	10.08	0.53
diet	No*	92.11	90.41	88.60	88.60	89.92	
Past conserv.	Mean	0.74	0.83	0.78	0.97	0.83	0.04
Behaviour [0,6]	SD	(0.95)	(1.00)	(0.91)	(1.04)	(0.98)	
Past conserv.	Mean	5.83	5.86	5.86	5.85	5.85	0.99
Beliefs [1,7]	SD	(0.99)	(0.95)	(0.96)	(0.97)	(0.97)	
Past animal cause	Mean	4.78	4.80	4.70	4.82	4.78	0.33
belief [1,7]	SD	(1.43)	(1.40)	(1.38)	(1.50)	(1.43)	
Sample size		266	271	272	272	1081	

(i) *p* values are from either non-parametric Kruskal–Wallis tests (for ordinal outcomes) or Chi squared tests (for categorical outcomes); (ii) Numbers in square brackets indicate the range of response scale i.e. Likert scales of 1 to 7 for ordinal outcomes, count of the number of past pro-conservation actions [0,6]; (iii) *Omitted categories in further analysis

In terms of past pro-conservation behaviours, 90% of the sample did not follow a vegetarian or vegan diet. In terms of civic actions like attending a protest, donating money/time etc., 47% had not taken up any of these actions, while 31% had taken up at least one action, and 17% had taken up at least two actions. Figure 3 in the Appendix shows the distribution of responses across groups.

Most people were found to hold pro-conservation beliefs with 20% at least somewhat agreeing and 75% agreeing (or strongly agreeing) that mass wildlife extinction and climate change are happening and are caused mostly by humans. There was relatively less agreement on whether coronavirus originated in animals with 30% of the sample at least somewhat agreeing with the animal origin story and just over 34% agreeing (or strongly agreeing). Figures 4 and 5 in the Appendix show the distribution of responses across groups.

Since the treatment was randomly allocated, most observable characteristics are evenly spread across treatment groups (as noted in the *p* values in Table 1). While the summary statistics of past pro-conservation behaviour don’t seem to show much difference across groups, the *p* value from the Kruskal–Wallis (balance) tests indicates some difference across groups (because a smaller share of those in the AHLC group relative to the control group reported undertaking no actions). As noted in Sect. 3.8 (and in the pre-registration plan), we will first conduct the main analysis without covariates and then add in the covariates in the subsequent analysis to address this issue.

Turning to pro-conservation outcomes in Table 2, the average donation was just under £8 (with the median and mode being £10, likely reflecting the fact that this was the default donation starting point). The mean donation in the control group is slightly lower at about £7.4 when compared to around £8.1 in the treatment groups. Overall, some 19% of the subjects chose not to donate while 81% donated some amount. When we examined the distribution of donation responses across groups (see Fig. 6 in Appendix), we found a slightly higher share of subjects donated £10 or over (i.e. default amount on the donations task) in the treatment groups: 53.9% in the AC narrative, 56.3% in the AHC narrative, and 51.8% in the AHLC narrative compared to 48.5% in the control group.

**Table 2:** Pro-conservation outcomes: Summary statistics by treatment groups

Outcome variables		Control		AC		AHC		AHLC		All		*p* value
Behaviour [£0, £20]	Donations	Mean	SD	Mean	SD	Mean	SD	Mean	SD	Mean	SD	
		7.35	5.88	8.06	6.04	8.19	5.96	8.12	6.15	7.93	6.01	0.34
Intentions [1, 5]	Protest	2.50	1.11	2.66	1.11	2.62	1.11	2.62	1.13	2.60	1.11	0.49
	Avoid meat	2.80	1.34	2.87	1.34	2.94	1.37	2.86	1.38	2.87	1.36	0.70
	Engagement	2.95	1.04	3.07	1.07	3.00	1.05	3.11	1.08	3.03	1.06	0.28
	Sustainable lifestyles	3.95	0.98	4.03	0.88	4.01	0.92	3.99	0.96	4.00	0.93	0.88
Policy support [1, 5]	Stricter regulation	4.44	0.80	4.53	0.76	4.57	0.73	4.51	0.71	4.51	0.75	0.21
	Meat tax	2.94	1.29	2.96	1.26	3.17	1.29	3.04	1.31	3.03	1.29	0.16
	Comm. Trade ban	3.98	1.01	4.10	0.99	4.24	0.95	4.14	0.97	4.12	0.98	0.01
	Conservation policies	3.79	0.82	3.86	0.76	3.99	0.78	3.90	0.78	3.89	0.79	0.02
Feelings of responsib.[1,5]	Personal	3.68	0.91	3.73	0.92	3.80	0.93	3.76	0.89	3.74	0.91	0.46
	Firms + Governments	4.40	0.76	4.45	0.75	4.56	0.70	4.49	0.72	4.48	0.73	0.03
Narrative engagement [1,5]	Mental	2.84	1.10	3.14	1.05	3.33	1.01	3.39	1.07	3.18	1.08	0.00
	Emotional	1.44	0.79	2.12	1.04	2.39	1.06	2.32	1.15	2.07	1.08	0.00
	Familiarity	1.20	0.56	3.20	1.25	2.93	1.24	3.11	1.21	2.62	1.37	0.00
Sample size		266.00		271.00		272.00		272.00		1081.00		

(i) *p* values are from non-parametric Kruskal–Wallis tests; (ii) Numbers in square brackets indicate the range of response scale i.e., up to £20 for donations, and Likert scales of 1 to 5 for the rest of the ordinal outcomes; (iii) The share of subjects who donated ≥ £10 (i.e. default amount on the donations task) were: 48.5% in the control group of, 53.9% in the AC narrative, 56.3% in the AHC narrative, 51.8% in the AHLC narrative, and 52.6% for the whole sample (see Figure A4 in Appendix). (iv) The conservation policies variable is an average of the other policy support variables

In terms of pro-conservation behavioural intentions, we found generally low average scores suggesting that people have relatively weak intentions to act in the specified pro-conservation ways in the future. The average score for intentions to engage in future social action, avoid eating meat, and engage with conservation organisations were 2.6, 2.9 and 3, respectively. The score for intentions to follow sustainable lifestyles was higher, averaging 4. Average scores were only slightly higher in the treated groups, relative to the control. Figures 7, 8, 9 and 10 in the Appendix present the distribution of responses across groups.

Support for the three wildlife conservation policies presented in the survey was relatively higher gaining average scores of around 4.5, 3 and 4.1 in support of stricter regulation of wildlife trade and farming, a meat tax, and banning commercial trade in wildlife, respectively. Once more, scores were slightly higher in the treated groups. Figures 11, 12, 13 and 14 in the Appendix present the distribution of responses across groups.

### Pro-conservation Outcomes

When we regressed the narrative treatment variable on donations, we found that the treatment coefficients were positive but the difference was not statistically significant when compared to the control group. The results are presented in Table 5 in the Appendix.

In Table 3, model (1) presents the results of the effect of treatment narratives on the likelihood of donating the default £10 amount and over (without any covariates). While all the treatment coefficients were positive, the difference was statistically significant at the 10% only for the AHC narrative relative to the control group. This result suggests that when exposed to the AHC narrative, subjects are 36.5% more likely to make a donation of £10 or higher compared to the control group.^10^

**Table 3:** Effect of narratives on support for conservation policies: Regression models without covariates

Regression models	(1)	(2)	(3)	(4)	(5)
Outcomes	Donation ≥ £10	Stricter regulation	Meat tax	Comm. trade ban	Conserv. policies
AC Narrative	1.240 (0.215)	1.289 (0.229)	1.037 (0.156)	1.281 (0.201)	1.165 (0.174)
AHC Narrative	1.365* (0.237)	1.418** (0.250)	1.374** (0.212)	1.679*** (0.267)	1.586*** (0.245)
AHLC Narrative	1.143 (0.197)	1.135 (0.194)	1.154 (0.181)	1.368** (0.214)	1.250 (0.193)
Constant	0.942 (0.116)				
Observations	1081	1081	1081	1081	1081
*r*^2^_p	0.00233	0.00224	0.00153	0.00398	0.00205
*p*	0.323	0.215	0.155	0.0121	0.0246
chi^2^	3.480	4.466	5.241	10.93	9.386

(i) Robust standard errors in parentheses; (ii) *** *p* < 0.01, ** *p* < 0.05, * *p* < 0.1; (iii) Model (1) presents log-odds coefficient from a logistic regression model, where the interpretation is an increase in the predicted log odds of the outcome (= 1) due to a one-unit increase in the predictor, holding everything else constant. Models (2) to (5) present ordered log-odds (logit) regression coefficients, where the interpretation is that for a one-unit increase in the predictor, the response variable level is expected to change by its respective regression coefficient in the ordered log-odds scale holding everything else constant; (iv) The control group is the omitted category

When we considered intentions to act in more pro-conservation ways, the regression results revealed that the narrative treatment coefficients are positive but not significant when compared to the control. Table 7 in the Appendix presents the results. Given the lack of robust treatment effects we focus the rest of our analysis on the effect of the narratives on support for different wildlife conservation policies.

Table 3 (models (2)–(5)) presents our main results where the narrative dummy is regressed on the policy support outcomes (without any covariates). Relative to the control group, the AHC narrative group elicited higher support for all policies and this difference was significant at the 5% level for stricter regulation and the meat tax, and at the 1% level for the wildlife trade ban and the aggregate conservation policies variable. More specifically, relative to the control group, being exposed to the AHC narrative increased the odds of a subject reporting higher support by one point for stricter regulation, the meat tax and the ban by 41.8%, 37.4% and 67.9% respectively. Exposure to the AHLC narrative significantly increased support only for the trade ban (at 5% level) when compared to the control group. This translated into an increase in the odds of a subject reporting higher support by one point for the ban by 36.8%.

These results indicate that exposure to the AHC narrative significantly increased pro-wildlife conservation policy support, in line with the possible existence of an outrage effect. Removing the human cause from the narrative (as in the AC narrative) or adding in an alternate causal explanation (as in the AHLC narrative) removes this effect. Overall, also considering the effects on donations and behavioural intentions, these results lend partial support to our first hypothesis.

Wald tests show that the difference in the AC and AHC coefficients was significant and positive (since AHC had a higher effect) at the 10% level for both the meat tax and the trade ban (chi^2^ = 3.56, *p* value = 0.06 and chi^2^ = 2.75, *p* value = 0.10 respectively). The difference between the AC and AHC coefficients was significant at the 5% level when the outcome was the aggregated support for conservation policies variable (chi^2^ = 4.38, *p* value = 0.04). There was no difference between the AC and AHLC coefficients, or AHC and AHLC coefficients in any of the models. These results suggest that adding the distal human cause in the AHC narrative increased pro-conservation policy support relative to the narrative that omits distal human cause (i.e. the AC narrative). This lends support for hypothesis two in the context of the wildlife policy outcome. We also found no difference between the AHC and AHLC narratives, and the AC and AHLC narratives, lending support to hypothesis three and four that there is no difference between these narratives in terms of their effect in the wildlife policy outcome.

We did not find any heterogeneous effects on policy support when we interacted a dummy for the narrative treatments with past pro-conservation behaviour, past pro-conservation beliefs and past beliefs about whether the virus originated in animals. The only exception was a positive and significant interaction effect between the AHLC and past beliefs about the animal cause of the virus (significant at the 5%) (see Table 9 in the Appendix).

**Fig. 2:**
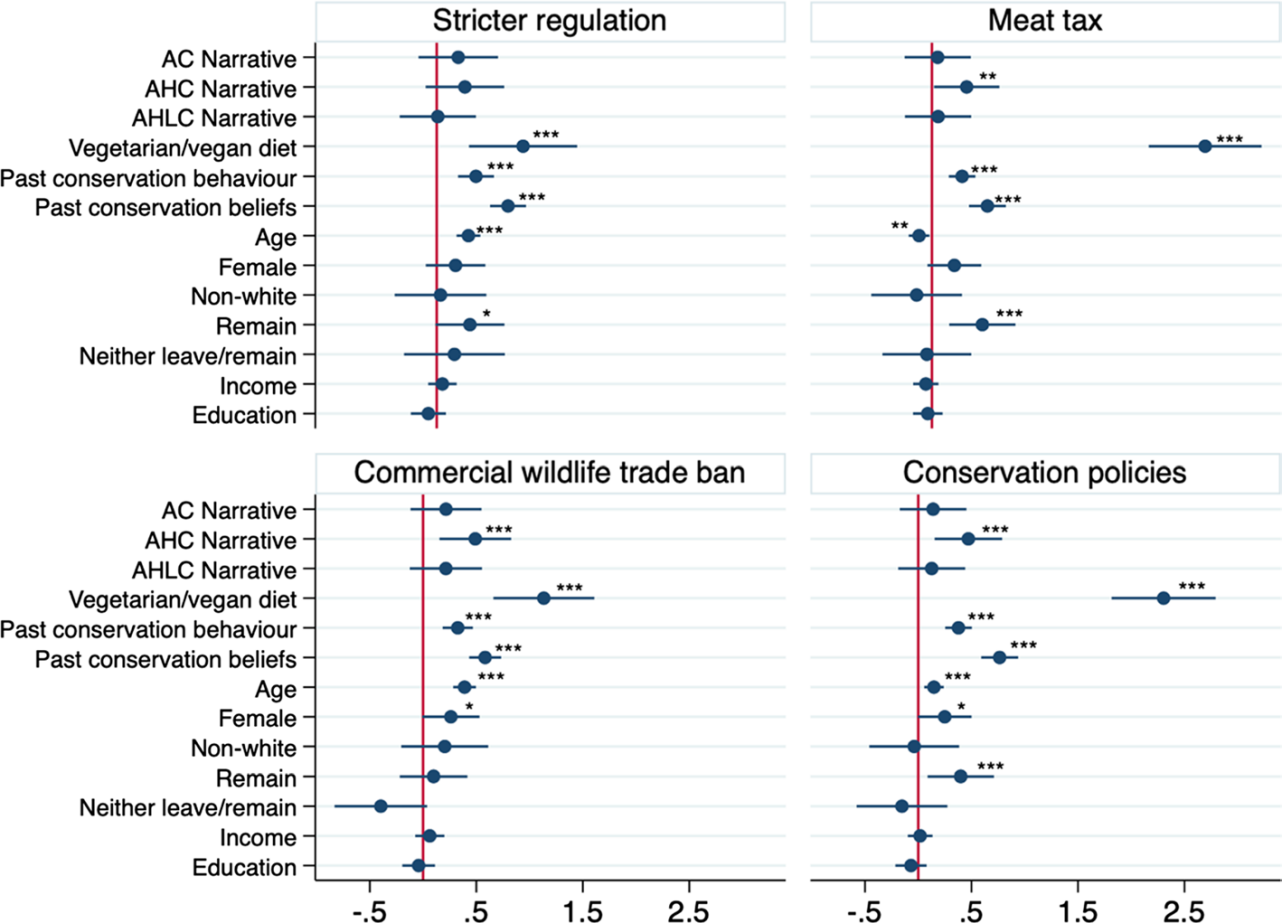
Effect of narratives on policy support: Ordered logistic regression coefficients with covariates. (i) ***p < 0.01, **p < 0.05, *p < 0.1; (ii) The figure presents the ordered log-odds (logit) regression coefficients and estimated confidence intervals at the 95% level. The coefficient interpretation is that for a one unit increase in the predictor, the response variable level is expected to change by its respective regression coefficient in the ordered log-odds scale holding everything else constant; (iii) Omitted categories are: Control group, Non-vegetarian/vegan diet, Not female, White and Leaver; (iv) Model is estimated with robust SE and is available in Table 8 in the Appendix

**Fig. 3:**
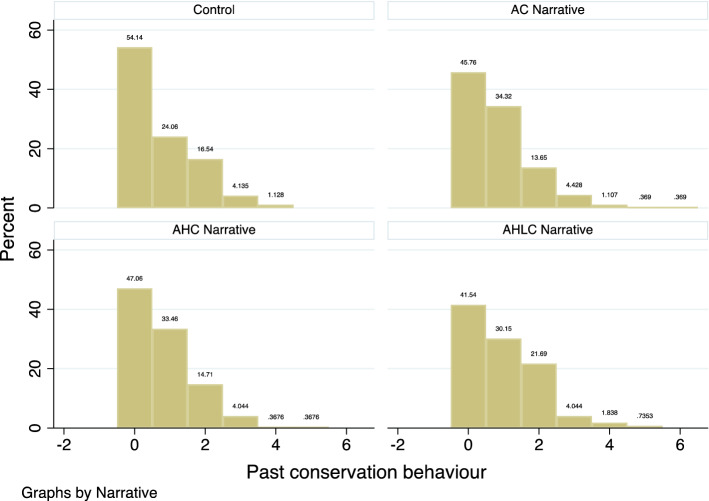
Distribution of past pro-conservation behaviour

**Fig. 4:**
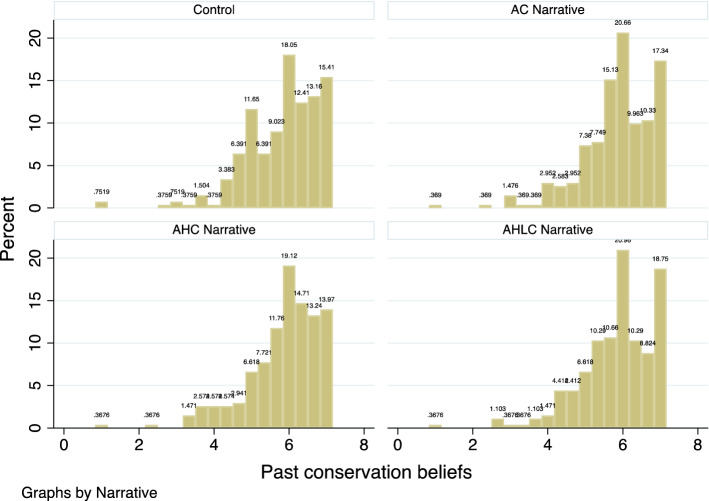
Distribution of past pro-conservation beliefs

**Fig. 5:**
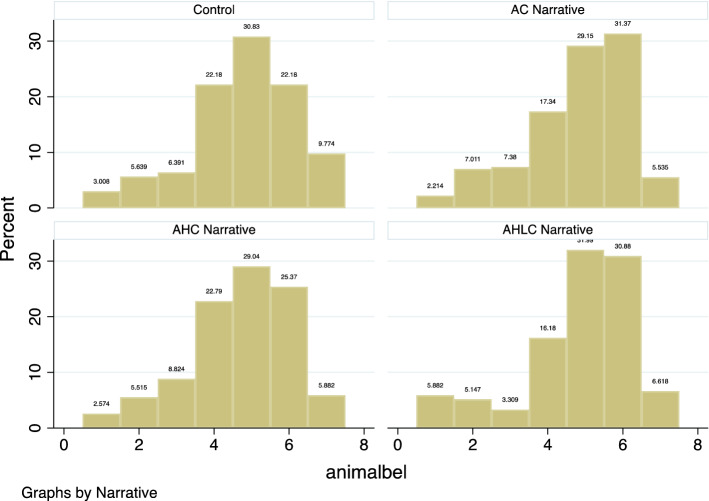
Distribution of past animal cause beliefs

**Fig. 6:**
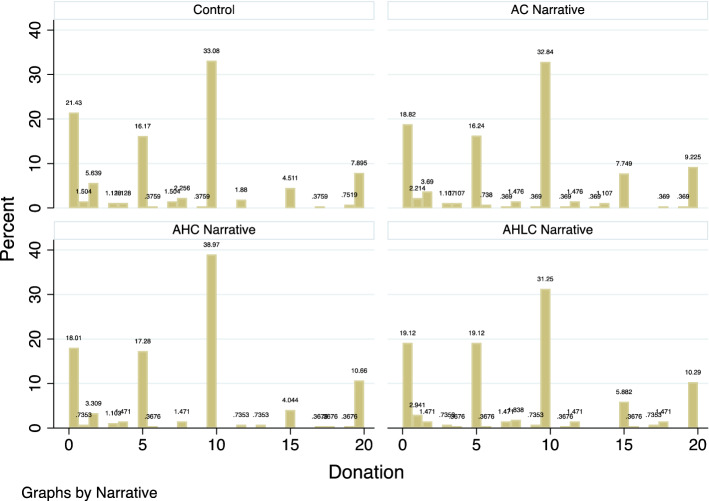
Distribution of donations

**Fig. 7:**
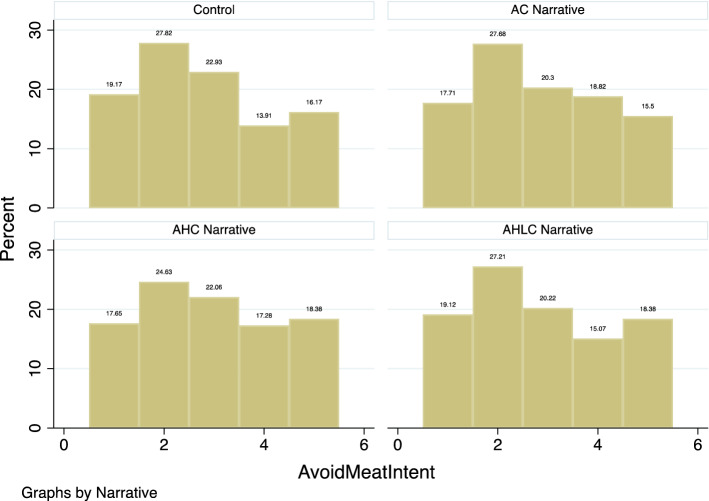
Distribution of intentions to avoid meat

**Fig. 8:**
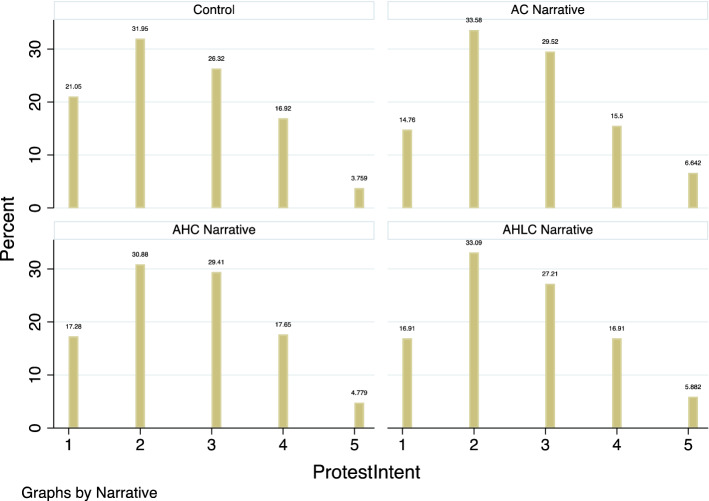
Distribution of intentions to protest

**Fig. 9:**
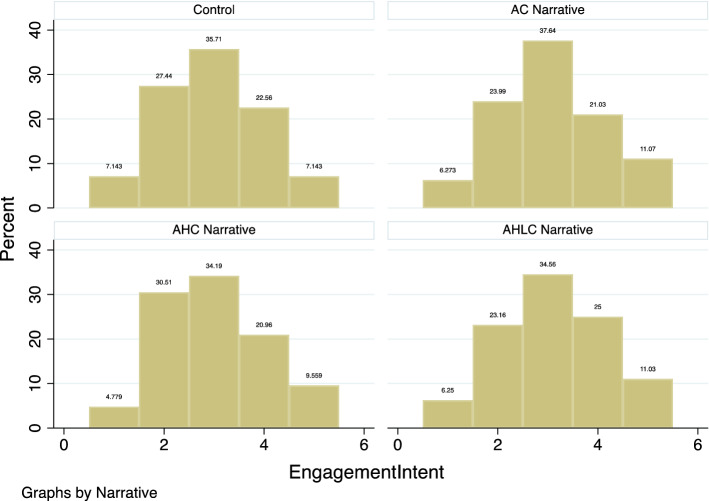
Distribution of intentions to engage with conservation organisations

**Fig. 10:**
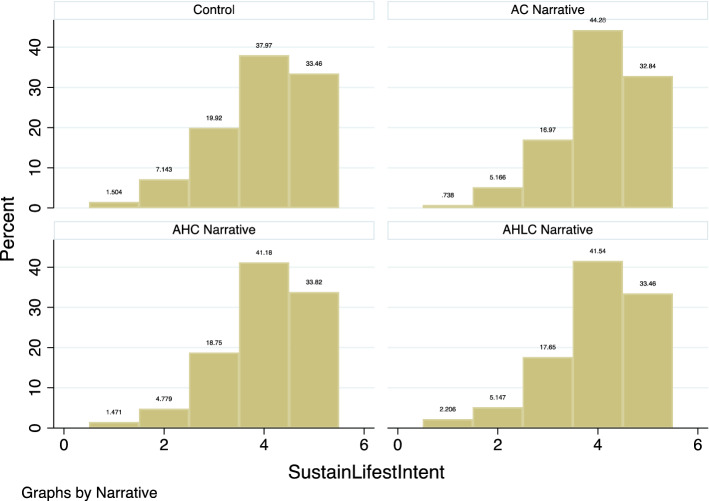
Distribution of intentions to have sustainable lifestyles

**Fig. 11:**
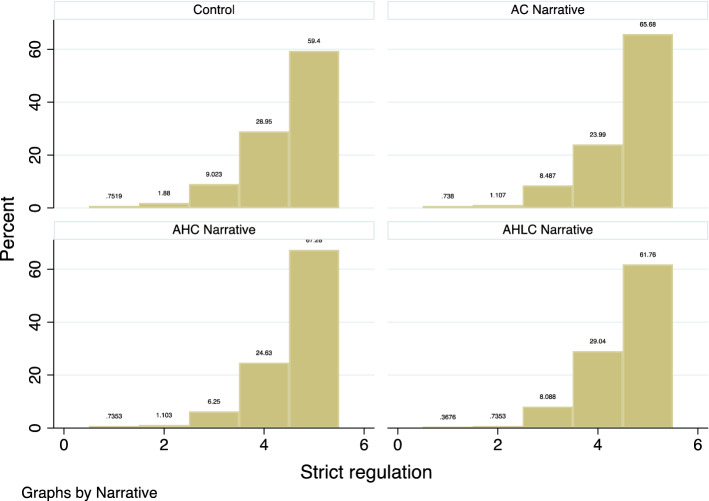
Distribution of support for stricter regulation

**Fig. 12:**
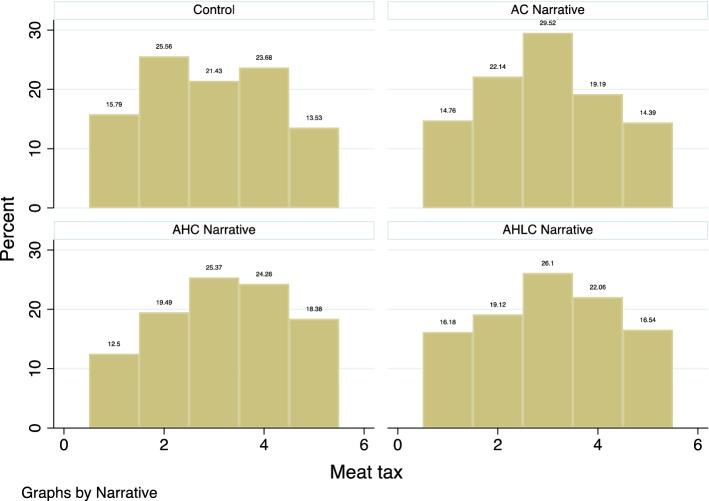
Distribution of support for meat tax

**Fig. 13:**
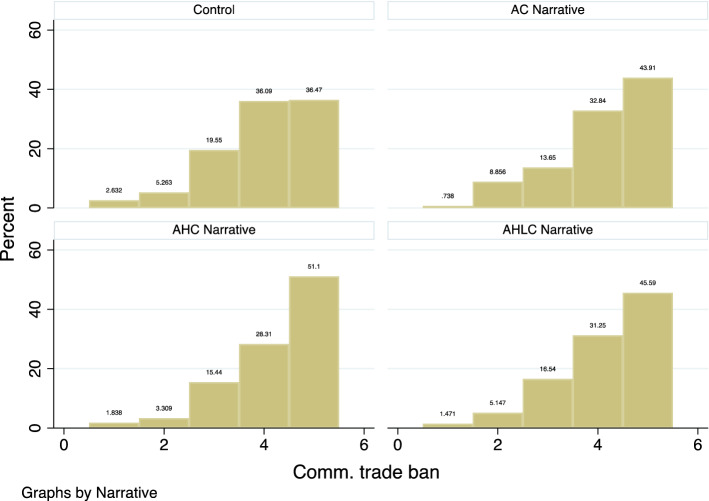
Distribution of support for ban

**Fig. 14:**
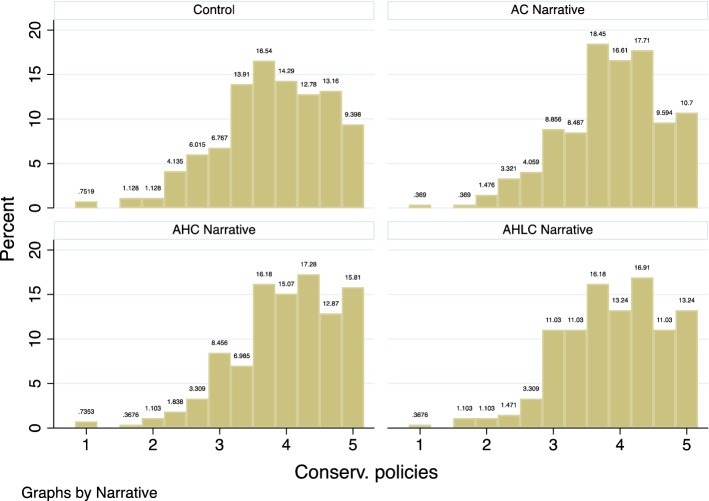
Distribution of support for conservation policies

Figure 2 plots the coefficients from the ordered logistic regression where the narrative dummy is regressed on the policy support outcomes with past conservation behaviour, past beliefs and socio-demographic covariates. The results are consistent in terms of effect size and significance level with the main results of the treatment effect presented in Table 3. The only exception is that the coefficient on the AHC narrative is no longer significant at the 5% level for the stricter regulation policy support. Following a vegetarian or vegan diet (relative to those who don’t), and past conservation behaviour and beliefs are the strongest predictors of policy support: the respective coefficients are all positive and statistically significant at the 1% level. Support for wildlife trade bans and stricter regulations (but not the meat tax) are positively associated with age. Compared to those identifying as Leavers, Remainers were more likely to support a meat tax (significant at 5%).

### Exploratory Analyses of Possible Mechanisms

Table 4 presents the results of the effect of the narrative treatments on felt responsibility and narrative engagement. While exposure to the treatment narratives increased feelings of personal responsibility, the difference was not significant (model 1). The AHC narrative, however, increased the feeling that firms and governments were responsible and the difference was positive and significant at the 1% level (model 2). In terms of covariates, stronger past pro-conservation beliefs and behaviours and following a vegetarian/vegan diet were positively associated with higher feelings of both personal and firms and government responsibility (significant mostly at the 1% level). Remainers felt firms and governments were more responsible compared to Leavers (significant at 1%).

**Table 4:** Effect on felt responsibility and narrative engagement: Ordered logistic regressions with covariates

Ordered logistic regressions	(1)	(2)	(3)	(4)	(5)
Outcomes	Feelings of responsibility		Narrative engagement		
	Personal	Firms & govt.	Mental	Emotional	Familiar
AC Narrative	1.022 (0.177)	1.188 (0.228)	1.693*** (0.285)	4.636*** (0.854)	57.755*** (14.392)
AHC Narrative	1.189 (0.209)	1.686*** (0.322)	2.276*** (0.382)	7.778*** (1.449)	36.217*** (8.698)
AHLC Narrative	1.001 (0.159)	1.198 (0.219)	2.534*** (0.438)	6.310*** (1.213)	46.815*** (11.118)
Vegetarian/vegan diet	2.564*** (0.525)	1.735** (0.449)	1.162 (0.217)	1.461* (0.322)	1.153 (0.246)
Past conservation behaviour	1.969*** (0.145)	1.626*** (0.147)	1.250*** (0.074)	1.452*** (0.102)	1.243*** (0.085)
Past conservation beliefs	1.634*** (0.127)	2.277*** (0.221)	1.273*** (0.097)	1.328*** (0.102)	1.082 (0.094)
Age	1.040 (0.052)	1.047 (0.059)	1.091* (0.049)	1.066 (0.056)	0.967 (0.047)
Female	1.172 (0.154)	0.691** (0.108)	1.556*** (0.197)	1.567*** (0.215)	0.615*** (0.083)
Non-white	0.678* (0.135)	0.803 (0.187)	0.850 (0.170)	1.251 (0.246)	0.687* (0.149)
Remain	0.979 (0.154)	1.812*** (0.294)	0.887 (0.131)	0.795 (0.133)	1.099 (0.171)
Neither Leave/Remain	0.705* (0.150)	0.867 (0.208)	0.759 (0.170)	0.765 (0.190)	1.208 (0.289)
Income	1.031 (0.066)	1.001 (0.070)	0.950 (0.054)	1.004 (0.063)	1.022 (0.069)
Education	1.121	1.016	1.035	0.915	1.247***
	(0.085)	(0.087)	(0.068)	(0.069)	(0.098)
Observations	1008	1008	1008	1008	1008
*p*	0	0	0	0	0
chi^2^	203.5	189.1	102.8	191.8	330.9

(i) Robust standard errors in parentheses; (ii) *** *p* < 0.01, ** *p* < 0.05, * *p* < 0.1; (iii) The table presents the ordered log-odds (logit) regression coefficients where the interpretation is that for a one unit increase in the predictor, the response variable level is expected to change by its respective regression coefficient in the ordered log-odds scale holding everything else constant; (iv) Omitted categories are: Control group, Non-vegetarian/vegan diet, Not female, White and Leaver

All the treatment narratives increased feelings of emotional and mental involvement in the article compared to the control group (significant at the 1% level) (models 3 and 4). Turning to whether effects were different between treatments, we found that the AHC narrative elicited higher mental and emotional involvement than the AC narrative (chi^2^ = 3.53, *p* value = 0.06 and chi^2^ = 10.50, *p* value = 0.00 respectively). The AHC narrative also elicited higher mental and emotional involvement than the AHLC narrative (chi^2^ = 6.33, *p* value = 0.01 and chi^2^ = 3.40, *p* value = 0.07 respectively). In terms of covariates, those with stronger past pro-conservation beliefs and behaviours reported feeling more engaged (significant at the 1% level).

Finally, unsurprisingly, subjects reported more familiarity with all the treatment narratives relative to the control narrative (about a Scottish castle). Subjects reported less familiarity with the AHC narrative when compared to the AC narrative (chi^2^ = 7.67, *p* value = 0.01) and also marginally less familiarity than with the AHLC narrative (chi^2^ = 2.70, *p* value = 0.10).

### Validity and Robustness Checks

To ensure that we controlled for any imbalance across groups in observable characteristics, we repeated the main analysis with the addition of covariates to find similar results, i.e. the AHC narrative increased the likelihood of donating £10 and over (significant at the 10% level), as well as support for conservation policies (significant at the 1% level), especially meat taxes (significant at the 5% level) and a wildlife trade ban (significant at 1% level) (Fig. 2, and Tables 6 and 8 in Appendix). These results were also similar when using ordinary least squares regressions instead of logistic and ordered logit models (for outcome of donations ≥ £10, and policy support variables respectively).

To check that the subjects read the narratives, we asked if they remembered the source of the article (i.e. the BBC). We also asked questions regarding the narrative content, namely whether the article discussed the cause of COVID-19 and if it mentioned other zoonotic diseases like the Swine flu. Reassuringly, 84% of subjects remembered the content and source of the narrative. Removing from the sample those who did not remember (reducing the sample size to 908) does not significantly alter the results: the regression analysis on the policy support outcomes and the likelihood of donating £10 and over showed the same results (Table 10 in the Appendix).

We also checked if our results were driven by prior attitudes and choices towards the BBC. Around 87.6% of the subjects used the BBC to obtain news online and this share was evenly distributed across treatment groups (chi^2^ = 1.01, *p* value = 0.80). Only 9.7% reported not trusting the BBC and again this proportion was spread evenly across groups (chi^2^ = 3.09, *p* value = 0.80). As before, the results on policy support are qualitatively similar after omitting these subjects (Table 11 in the Appendix).

In addition, we checked if the positive effect of the AHC narrative on support for stricter regulation, meat taxes, wildlife trade bans and aggregate conservation policies (i.e. outcome averaging all three policies), and the likelihood of donation ≥ £10 (when compared to the control group), was replicated after adjusting for hypothesis testing with multiple outcomes following List et al. (2019). The differences in means between the AHC and control group remained significant at the 10% level for the donation ≥ £10 dummy, and at the 1% level for the commercial wildlife trade ban and the overall conservation policy variables. The adjusted *p* value fell to 10% from the 5% level for stricter regulation and meat taxes from the main regression results in Table 3 when using List et al. (2019)’s correction method (see Table 12 in the Appendix). Overall this suggests that the effect on the aggregated policy support variable and commercial wildlife trade bans are the most robust.

We also explored whether there were differences between groups in the time people spent reading narratives. The summary statistics are similar across the groups, namely 75 s for the Control group (S.D. = 51.6), 65 s for the AC group (S.D. = 72.9), 76 s for the AHC group (S.D. = 53.5), and 77 s for the AHLC group (S.D. = 99) (for all groups, the pooled average is 73 s (S.D. = 72)). We ran an OLS regression model to test whether the differences were significant (see Table 13 in Appendix). Time spent was marginally lower by 10 s in the AC group (significant at 10%, results below). Wald tests showed that differences between the AC and AHC group were significantly different at the 5% level (chi^2^ = 4.19, *p* value = 0.04). Differences between the AC and AHLC, and AHC and AHLC were not significant. In sum, participants conformed with expectations by spending marginally less time on the shorter AC narrative.

When we checked why people chose not to donate, the most commonly cited reason was ‘I need to save money for myself and for my family’ (37.7% of those choosing not to donate), followed by ‘I already donate enough’ (13.9%) and ‘I’d rather keep the money’ (10.3%). These reasons were evenly distributed across groups. Some 7.6% and 4% of the subjects (around 26 out of 1080) people reported not liking or knowing the charities on the list (this was spread evenly across groups as well), despite our effort to offer people a wide choice of environmental charities. Upon examining the written comments, we found that some subjects preferred to donate to health causes, which may reflect the current preoccupations with health in the COVID-19 context.

Another concern was that subjects might have guessed the true objective of the experiment and in doing so might have changed their responses. On the one hand, social desirability bias predicts that subjects may increase their pro-conservation outcomes if they think that the causal information in the articles was designed for that purpose. On the other hand, if subjects perceive an intent to manipulate, narrative-format communications can induce reactance and any effects may be reduced (Escalas 2007). It is not clear which of these effects dominate in this study. We attempted to address this by using filler questions and framing the title of the survey experiment in a neutral way.

Lastly, while we do observe a higher share of people donating at or above the default of £10 in the AHC narrative, otherwise we do not observe robust effects on donations or intentions to act in more pro-conservation ways. One explanation for this may be that the narratives we tested were not ‘strong’ enough (for e.g. compared to videos) or the exposure was not long enough (e.g. repeated in many ways over a long period) to have an effect on behaviour even if it affected policy preferences. Another possibility is that our sample size is not large enough to detect the true effects on donation and intentions, which may be smaller. It is also possible that effects are smaller at this point in time because of the broader COVID-19 context, which may have shifted people’s focus and inclination to take prosocial actions to improve health rather than environmental outcomes. That said, we believe this study is a first step in the right direction since we have a higher-powered experiment with a comparable active control group when compared to most existing research. Future work can examine the impact of such narratives on a larger and more representative sample to address some of the limitations of this study.

## Discussion and Conclusion

We examined the effects of three alternative narratives currently in circulation, each proposing different causal explanations for COVID-19, on pro-wildlife conservation outcomes. The AC narrative points to origins amongst wild animals, the AHC narrative includes the causal link with the human depletion of nature, and the AHLC narrative adds the possibility of blame on a biosecurity lab.

We found that the AHC narrative influenced people’s willingness to support conservation policies, especially commercial wildlife trade bans. This is in line with both the outrage effect (Kahneman et al. 1993; Bulte et al. 2005; Shreedhar and Mourato 2019) and the predicted impact of responsibilizing narratives (Bénabou et al. 2018). We found that the AHC narrative increased the feeling that firms and governments were responsible, but had no significant effect on personal responsibility, which is only partially in line with the responsibility effect (Walker et al. 1999). One possible explanation is that people may not feel the ‘burden’ of personal responsibility when they are not solely responsible for an outcome and there are multiple are other parties who are also responsible (Cryder and Loewenstein 2012). This may give them some more ‘moral wiggle room’ (Dana et al. 2007) but not necessarily dampen their outrage at others they perceive are guilty. The increased feeling that firms and governments are responsible may explain why subjects supported policies that ostensibly affected these other parties (e.g. bans).

But the findings also suggest that the effect of narrative exposure is fragile: either removing information about the human cause (as in the AC group), or conversely adding a counter-narrative (as in the AHLC group) can attenuate these effects. That AC is less effective than AHC is compatible with the explanation that natural-causes elicit lower responses than human-caused problems (Brown et al. 2005; Bulte et al. 2005). That AHLC is less effective than AHC is compatible with the prediction that absolving narratives dampen prosocial behaviour (Bénabou et al. 2018). It also supports findings that even brief exposure to climate conspiracy theories can dampen pro-climate actions (Greitemeyer 2013; van der Linden 2015). Moreover, it aligns with literature suggesting that articles presenting ‘duelling experts’ without any sense of how the weight of evidence is distributed generates greater climate scepticism and is a barrier to engagement (Corbett and Durfee 2004; Lorenzoni et al. 2007; Corner et al. 2012).

Understanding the impact of narratives shared on online news and social media is crucial, since this is where people increasingly get information about public health and environmental issues (Dahlstrom 2014; Pearson et al. 2016). It is also an important route to forming preferences over policy issues. We found that the AHC narrative linking human destruction of nature to COVID-19 increases support for wildlife conservation policies. Interestingly, this narrative was rated as the least familiar by subjects although it is the story favoured by environmental policymakers. The results from this experiment suggest that there is scope to use this narrative to grow public engagement with extinction. This public support is key to craft a durable and legitimate long-term policy response to COVID-19, which concurrently addresses anthropogenic mass wildlife extinction.

## Appendix

See Tables 5, 6, 7, 8, 9, 10, 11, 12, 13 and Figs. 3, 4, 5, 6, 7, 8, 9, 10, 11, 12, 13, 14.

**Table 5:** Effect of narratives on charitable donations: Cragg-Hurdle model without covariates

Cragg-Hurdle regression	(1)	(2)
Outcome: Donation	Probability	Amount
AC Narrative	1.097 (0.135)	2.041 (1.265)
AHC Narrative	1.131 (0.140)	2.209 (1.351)
AHLC Narrative	1.085 (0.133)	2.362 (1.474)
Constant	2.207*** (0.190)	5294.980*** (2384.880)
Observations	1081	1081
*p*	0.487	0.487
chi^2^	2.437	2.437

(i) Robust standard errors in parentheses (ii) *** *p* < 0.01, ** *p* < 0.05, * *p* < 0.1 (iii) The table presents the regression coefficients from the linear Cragg–Hurdle model: the probability of donating was estimated using a Probit regression model (so the lower hurdle was £0 or the decision to donate any amount) and a Truncated-linear regression model was used to estimated effects on the amount donated, conditional on having decided to make any donate (iv) Omitted category is the control group

**Table 6:** Effect of narratives on the likelihood of donating £10 and over: Logistic regression model with covariates

Logistic regression models	(1)	(2)
Outcome	Donation $\ge$ £10	
AC Narrative	1.240 (0.215)	1.225 (0.233)
AHC Narrative	1.365* (0.237)	1.365* (0.255)
AHLC Narrative	1.143 (0.197)	1.032 (0.189)
Vegetarian/vegan diet		1.214 (0.283)
Past conservation behaviour		1.346*** (0.108)
Past conservation beliefs		1.206** (0.096)
Age		1.110* (0.061)
Female		1.922***
		(0.277)
Non-white		0.820 (0.186)
Remain		1.167 (0.197)
Neither L/R		1.078 (0.279)
Income		1.103 (0.077)
Education		1.083 (0.088)
Constant	0.942 (0.116)	0.067*** (0.038)
Observations	1081	1008
*p*	0.323	4.76e − 09
chi^2^	3.480	65.78

(i) Robust standard errors in parentheses; (ii) *** *p* < 0.01, ** *p* < 0.05, * *p* < 0.1; (iii) Models (1) and (2) log-odds coefficient from a logistic regression model, which indicate the amount of increase in the predicted log odds of the outcome = 1 due to a one-unit increase in the predictor, holding everything else constant; (iv) Omitted categories are: Control group, Non-vegetarian/vegan diet, Not female, White and Leaver

**Table 7:** Treatment effect of narrative on behavioural intentions: Ordered logistic model without covariates

Ordered logistic regression	(1)	(2)	(3)	(4)
Outcome	Protest/social action	Avoid meat	Engagement in conservation	Sustainable lifestyles
AC Narrative	1.264 (0.196)	1.092 (0.164)	1.199 (0.185)	1.132 (0.181)
AHC Narrative	1.209 (0.190)	1.200 (0.183)	1.051 (0.162)	1.108 (0.181)
AHLC Narrative	1.189 (0.188)	1.076 (0.166)	1.314* (0.205)	1.086 (0.178)
Observations	1081	1081	1081	1081
*p*	0.465	0.695	0.282	0.882
chi^2^	2.556	1.445	3.812	0.663

(i) Robust standard errors in parentheses; (ii) *** *p* < 0.01, ** *p* < 0.05, * *p* < 0.1; (iii) The table presents the ordered log-odds (logit) regression coefficients where the interpretation is that for a one unit increase in the predictor, the response variable level is expected to change by its respective regression coefficient in the ordered log-odds scale holding everything else constant; (iv) Omitted category is the control group

**Table 8:** Treatment effect of narratives on support for conservation policies: Ordered logistic models with covariates

Ordered logistic regression	(1)	(2)	(3)	(4)
Outcome	Stricter regulation	Meat tax	Comm. trade ban	Conservation. policies
AC Narrative	1.234	1.058	1.240	1.154
	(0.245)	(0.175)	(0.211)	(0.182)
AHC Narrative	1.317	1.406**	1.633***	1.597***
	(0.258)	(0.229)	(0.280)	(0.256)
AHLC Narrative	1.010	1.063	1.239	1.131
	(0.192)	(0.176)	(0.214)	(0.181)
Vegetarian/vegan diet	2.327***	14.478***	3.108***	8.494***
	(0.628)	(4.076)	(0.751)	(2.151)
Past conservation behaviour	1.468***	1.344***	1.385***	1.460***
	(0.132)	(0.089)	(0.100)	(0.092)
Past conservation beliefs	2.009***	1.722***	1.791***	2.176***
	(0.180)	(0.158)	(0.137)	(0.188)
Age	1.364***	0.882**	1.476***	1.224***
	(0.081)	(0.045)	(0.080)	(0.056)
Female	1.202	1.246	1.299*	1.270*
	(0.179)	(0.167)	(0.178)	(0.162)
Non-white	1.038	0.862	1.225	1.012
	(0.237)	(0.195)	(0.256)	(0.217)
Remain	1.384*	1.638***	1.104	1.453**
	(0.238)	(0.271)	(0.179)	(0.226)
Neither leave/remain	1.189	0.952	0.672*	0.867
	(0.299)	(0.211)	(0.149)	(0.187)
Income	1.057	0.943	1.065	1.032
	(0.075)	(0.060)	(0.074)	(0.060)
Education	0.921	0.961	0.959	0.936
	(0.081)	(0.071)	(0.075)	(0.069)
Observations	1008	1008	1008	1008
*p*	0	0	0	0
chi^2^	144.8	272.5	181.4	304.3

(i) Robust standard errors in parentheses (ii) *** *p* < 0.01, ** *p* < 0.05, * *p* < 0.1 (iii) The table presents the ordered log-odds (logit) regression coefficients where the interpretation is that for a one unit increase in the predictor, the response variable level is expected to change by its respective regression coefficient in the ordered log-odds scale holding everything else constant (iv) Omitted categories are: Control group, Non-vegetarian/vegan diet, Not female, White and Leaver

**Table 9:** Interaction effect of narratives with past beliefs about origin of coronavirus from animals: Ordered logistic models without covariates

Ordered logistic regression model	(1)
Outcome	Meat tax
AC Narrative	0.809
	(0.436)
AHC Narrative	1.445
	(0.800)
AHLC Narrative	0.378*
	(0.197)
Past animal cause belief	1.037
	(0.083)
AC Narrative x Past animal cause belief	1.054
	(0.116)
AHC Narrative x Past animal cause belief	0.991
	(0.111)
AHLC Narrative x Past animal cause belief	1.263**
	(0.132)
Observations	1081
*p*	0.00160
chi^2^	23.15

(i) Robust standard errors in parentheses (ii) *** *p* < 0.01, ** *p* < 0.05, * *p* < 0.1 (iii) The table presents the ordered log-odds (logit) regression coefficients where the interpretation is that for a one unit increase in the predictor, the response variable level is expected to change by its respective regression coefficient in the ordered log-odds scale holding everything else constant (iv) Omitted category is the control group

**Table 10:** Effect of narrative on outcomes: sample who didn’t forget information in the treatments

Regression models	(1)	(2)	(3)	(4)	(5)
Dependent variables	Donation $\ge$ £10	Stricter regulation	Meat tax	Comm. trade ban	Conserv. policies
AC Narrative	1.359	1.337	1.147	1.192	1.210
	(0.258)	(0.261)	(0.189)	(0.203)	(0.198)
AHC Narrative	1.408*	1.479**	1.462**	1.718***	1.677***
	(0.264)	(0.281)	(0.246)	(0.293)	(0.274)
AHLC Narrative	1.101	1.074	1.176	1.404**	1.267
	(0.206)	(0.197)	(0.195)	(0.237)	(0.206)
Constant	0.933				
	(0.123)				
Observations	908	908	908	908	908
p	0.203	0.139	0.158	0.0123	0.0168
chi2	4.609	5.492	5.197	10.90	10.22

(i) Robust standard errors in parentheses; (ii) *** *p* < 0.01, ** *p* < 0.05, * *p* < 0.1; (iii) Model (1) presents log-odds coefficient from a logistic regression model, where the interpretation is an increase in the predicted log odds of the outcome (= 1) due to a one-unit increase in the predictor, holding everything else constant. Models (2) to (5) present ordered log-odds (logit) regression coefficients, where the interpretation is that for a one-unit increase in the predictor, the response variable level is expected to change by its respective regression coefficient in the ordered log-odds scale holding everything else constant; (iv) The control group is the omitted category

**Table 11:** Effect of narrative on outcomes: sub-sample trusting the BBC

Regression models	(1)	(2)	(3)	(4)	(5)
Dependent variables:	Donation $\ge$ £10	Stricter regulation	Meat tax	Comm. trade ban	Conserv. policies
AC Narrative	1.205	1.351	1.069	1.256	1.204
	(0.218)	(0.251)	(0.169)	(0.207)	(0.189)
AHC Narrative	1.305	1.451**	1.410**	1.671***	1.626***
	(0.238)	(0.269)	(0.233)	(0.280)	(0.265)
AHLC Narrative	1.169	1.172	1.247	1.457**	1.353*
	(0.212)	(0.209)	(0.203)	(0.239)	(0.215)
Constant	0.960				
	(0.123)				
Observations	976	976	976	976	976
*p*	0.522	0.190	0.148	0.0147	0.0235
chi^2^	2.251	4.765	5.348	10.52	9.484

(i) Robust standard errors in parentheses; (ii) *** *p* < 0.01, ** *p* < 0.05, * *p* < 0.1; (iii) Model (1) presents log-odds coefficient from a logistic regression model, where the interpretation is an increase in the predicted log odds of the outcome (= 1) due to a one-unit increase in the predictor, holding everything else constant. Models (2) to (5) present ordered log-odds (logit) regression coefficients, where the interpretation is that for a one-unit increase in the predictor, the response variable level is expected to change by its respective regression coefficient in the ordered log-odds scale holding everything else constant; (iv) The sub-sample includes those reporting ‘Yes’ and ‘Maybe’ to trusting the BBC (v) The control group is the omitted category

**Table 12:** Multiple Hypothesis Test check from List et al. (2019)

Outcome	Diff. in	Unadj p-value	Adj p-value		
	means	R3.1	T3.1	Bonferroni	Holm
Donation ≥ £10	0.08	0.07	0.07	0.58	0.07
Stricter regulation	0.12	0.06	0.06	0.48	0.06
Meat tax	0.23	0.04	0.08	0.31	0.08
Comm. Trade ban	0.25	0.00	0.01	0.02	0.01
Consv policies	0.20	0.00	0.01	0.02	0.01

Diff. in means = Difference in means between the AHC treatment and control groups. The *p* values are adjusted for multiple outcomes and is estimated from stata command ‘mhtexp’ with bootstrap standard errors (3000 reps)

**Table 13:** Time spent on narrative page: Ordinary Least Squares (OLS) regression models

OLS Regression model:	(1)
Dependent variables:	Time on page
AC Narrative	− 10.19
	(5.44)
AHC Narrative	1.04
	(4.53)
AHLC Narrative	1.723
	(6.79)
Constant	75.05
	(3.16)
Observations	1081
R-squared	0.00
F	1.66

(i) Robust standard errors in parentheses; (ii) *** *p* < 0.01, ** *p* < 0.05, * *p* < 0.1

## Footnotes

1 In a news article published on 05 June 2020 in the journal Nature, Cyranoski (2020) provides the most recent review of the evidence for the animal versus lab origin of the virus at the time of writing. Currently available genome sequencing studies indicate the SARS-CoV-2 shares 96% of its genetic sequence with a group of viruses found in horseshoe bats (which can be found in Yunnan in China). Cyranoski noted that determining whether the lab was responsible for COVID-19 will require a forensic investigation for viruses that match the genetic sequence of SARS-CoV-2, and evidence that it could have escaped (though lab samples, staff interviews, review of lab books and records of safety incidents etc.). There have been no records of any accidents or lab workers falling ill so far, and scientists at the lab have refuted suggestions that the lab has ever had a virus similar to SARS-CoV-2. Finding a virus nearly identical to SARS-CoV-2 in an animal would provide the most persuasive evidence for how it passed to people, and would require extensive sampling of coronaviruses in wildlife and livestock in China.

2 The OSF preregistration can be found at this link.

3 The effect size was chosen based on Braddock and Dillard (2016)’s meta-analyses which found small to medium positive correlation effects of narratives on behaviour (0.23) and when using non-fiction narratives (0.25).

4 To illustrate, the lab story was adapted from Mallapaty (2020), a narrative-format article in the journal Nature which discussed the source of the coronavirus. The original text read: “US President Donald Trump has fuelled suggestions that the virus might have leaked from a laboratory in Wuhan, where the outbreak started. There is no evidence for that claim.” We found this to be a succinct way of presenting the lab story, but we modified the text by replacing words like “fuelled” and “leaked” with more neutral words like “suggested” and “came”.

5 We included both UK and international nature conservation and animal welfare charities: Extinction Rebellion, Royal Society for the Protection of Birds (RSPB), World Wide Fund for Nature (WWF), International Union for Conservation of Nature (IUCN), Royal Society for the Prevention of Cruelty to Animals (RSPCA). To mitigate any differences in perceived quality between organisations, we mentioned that all these charities were registered with the Fundraising Regulator. .

6 We tried to mitigate a possible windfall effect (of receiving £20 on top of the participation fee) (Carlsson et al. 2013) by stating in the instructions that the possibility of winning was linked to fully completing the survey. It is possible that the low probability of winning the pay-out may inflate giving, although other papers do not find such effects (Charness et al. 2016). While we tried our best to achieve some balance between offering a more realistic setting and stakes in the donations task, we cannot rule out that factors such as the method of payment and charity list affect our results. These design factors should be kept in mind while interpreting the results.

7 We measured other variables which were not used in the analysis and the full list can be obtained via the OSF preregistration form.

8 The Cragg Hurdle model was estimated using the ‘churdle’ command in Stata 15. The treatment effect on the probability of donating was estimated using a Probit regression model (so the lower hurdle was £0 or the decision to donate any amount), and a Truncated-linear regression model was used to estimated effects on the amount donated, conditional on having decided to make a donation.

9 The ordered logistic model was estimated using the ‘ologit’ command on Stata 15. Postestimation tests (using the Brant test of parallel regression assumption via the ‘brant’ command) revealed that the proportional odds assumption was not violated.

10 Wald tests show that differences between the treatment indicators are not statistically significant. The AHC narrative remains positive and statistically significant at the 10% level when we added covariates to the regression model, as shown in Table 6 in the Appendix. Amongst the covariates, past pro-conservation behaviour, beliefs and being female positively predicted the likelihood of subjects donating £10 and over (at the 1% or 5% level). .

## References

[CR1] Andersen KG, Rambaut A, Lipkin WI (2020). The proximal origin of SARS-CoV-2. Nat Med.

[CR2] Appel M, Gnambs T, Richter T, Green MC (2015). The Transportation scale-short form (TS–SF). Media Psychol.

[CR3] Aust F, Diedenhofen B, Ullrich S, Musch J (2013). Seriousness checks are useful to improve data validity in online research. Behav Res.

[CR4] Bateman TS, O’Connor K (2016). Felt responsibility and climate engagement: distinguishing adaptation from mitigation. Global Environ Chang.

[CR5] BBC (2020) “Unique” triangular castle captured in 3D. BBC News. https://www.bbc.co.uk/news/uk-scotland-south-scotland-52722380#:~:text=A%203D%20model%20has%20been,%22unique%20among%20British%20castles%22.&text=Caerlaverock%20Castle%2C%20in%20Dumfries%20and,%22pleasing%20geometry%22%20during%20lockdown. Accessed on 05 June 2020

[CR6] Beaumont P (2020) Where did Covid-19 come from? What we know about its origins. In: The Guardian. http://www.theguardian.com/world/2020/may/01/could-covid-19-be-manmade-what-we-know-about-origins-trump-chinese-lab-coronavirus. Accessed 12 June 2020

[CR7] Bénabou R, Falk A, Tirole J (2018) Narratives, imperatives, and moral reasoning. NBER Working Paper No. 24798, National Bureau of Economic Research

[CR8] Böhm G, Pfister H-R (2017). The perceiver’s social role and a risk’s causal structure as determinants of environmental risk evaluation. J Risk Res.

[CR9] Boyd C (2020) Deadly coronavirus outbreak DID start at Wuhan animal market | Daily Mail Online. https://www.dailymail.co.uk/health/article-7935167/Deadly-coronavirus-outbreak-DID-start-animal-market-Wuhan-tests-confirm.html. Accessed 12 June 2020

[CR10] Braddock K, Dillard JP (2016). Meta-analytic evidence for the persuasive effect of narratives on beliefs, attitudes, intentions, and behaviors. Commun Monogr.

[CR11] Briggs H (2020) Coronavirus: Putting the spotlight on the global wildlife trade. BBC News. https://www.bbc.co.uk/news/science-environment-52125309. Accessed 12 June 2020

[CR12] Brown TC, Peterson GL, Brodersen RM (2005). The judged seriousness of an environmental loss is a matter of what caused it. J Environ Psychol.

[CR13] Bruner J (1991). The Narrative Construction of Reality. Crit Inq.

[CR14] Bulte E, Gerking S, List JA, De Zeeuw A (2005). The effect of varying the causes of environmental problems on stated WTP values: evidence from a field study. J Environ Econ Manag.

[CR15] Carlsson F, He H, Martinsson P (2013). Easy come, easy go. Exp Econ.

[CR16] Carrington D (2020a) Coronavirus: ‘Nature is sending us a message’, says UN environment chief. The Guardian. https://www.theguardian.com/world/2020/mar/25/coronavirus-nature-is-sending-us-a-message-says-un-environment-chief. Accessed 12 June 2020

[CR17] Carrington D (2020b) Halt destruction of nature or suffer even worse pandemics, say world’s top scientists. The Guardian. http://www.theguardian.com/world/2020/apr/27/halt-destruction-nature-worse-pandemics-top-scientists. Accessed 12 June 2020

[CR18] Charness G, Gneezy U, Halladay B (2016). Experimental methods: pay one or pay all. J Econ Behav Organ.

[CR19] Chater N, Loewenstein G (2016). The under-appreciated drive for sense-making. J Econ Behav Organ.

[CR20] Cooper KE, Nisbet EC (2016). Green narratives: how affective responses to media messages influence risk perceptions and policy preferences about environmental hazards. Sci Commun.

[CR21] Corbett JB, Durfee JL (2004). Testing public (un)certainty of science: media representations of global warming. Sci Commun.

[CR22] Corner A, Whitmarsh L, Xenias D (2012). Uncertainty, scepticism and attitudes towards climate change: biased assimilation and attitude polarisation. Clim Change.

[CR23] Cryder CE, Loewenstein G (2012). Responsibility: the tie that binds. J Exp Soc Psychol.

[CR24] Cyranoski D (2020) The biggest mystery: what it will take to trace the coronavirus source. Nature. https://www.nature.com/articles/d41586-020-01541-z. Accessed 29 June 2020 10.1038/d41586-020-01541-z 32504020

[CR25] Dahlstrom MF (2010). The role of causality in information acceptance in narratives: an example from science communication. Commun Res.

[CR26] Dahlstrom MF (2014). Using narratives and storytelling to communicate science with nonexpert audiences. PNAS.

[CR27] Dana J, Weber RA, Kuang JX (2007). Exploiting moral wiggle room: experiments demonstrating an illusory preference for fairness. Econ Theory.

[CR28] Day B, Bateman IJ, Carson RT (2012). Ordering effects and choice set awareness in repeat-response stated preference studies. J Environ Econ Manag.

[CR29] Dolan P (2019). Happy ever after: escaping the myth of the perfect life.

[CR30] Eitel A, Scheiter K (2015). Picture or text first? Explaining sequence effects when learning with pictures and text. Educ Psychol Rev.

[CR31] Eliaz K, Spiegler R (2018) A model of competing narratives. https://arxiv.org/pdf/1811.04232.pdf. Accessed 12 June 2020

[CR32] Escalas JE (2007). Self-referencing and persuasion: narrative transportation versus analytical elaboration. J Consum Res.

[CR33] Faul F, Erdfelder E, Lang A-G, Buchner A (2007). G*Power 3: a flexible statistical power analysis program for the social, behavioral, and biomedical sciences. Behav Res Methods.

[CR34] Gardener B (2020) Exclusive: Coronavirus began “as an accident” in Chinese lab, says former MI6 boss. https://www.telegraph.co.uk/news/2020/06/03/exclusive-coronavirus-began-accident-disease-escaped-chinese/. Accessed 12 June 2020

[CR35] Gifford R, Nilsson A (2014). Personal and social factors that influence pro-environmental concern and behaviour: a review. Int J Psychol.

[CR36] Green MC, Brock TC (2000). The role of transportation in the persuasiveness of public narratives. J Pers Soc Psychol.

[CR37] Green MC, Brock TC, Kaufman GF (2004). Understanding media enjoyment: the role of transportation into narrative worlds. Commun Theory.

[CR38] Greitemeyer T (2013). Beware of climate change skeptic films. J Environ Psych.

[CR39] Hockley WE, Bancroft T (2011). Extensions of the picture superiority effect in associative recognition. Can J Exp Psychol.

[CR40] Kahneman D, Ritov I (1994). Determinants of stated willingness to pay for public goods: a study in the headline method. J Risk Uncertain.

[CR41] Kahneman D, Ritov I, Jacowitz KE, Grant P (1993). Stated willingness to pay for public goods: a psychological perspective. Psychol Sci.

[CR42] Kahneman D, Schkade D, Sunstein C (1998). Shared outrage and erratic awards: the psychology of punitive damages. J Risk Uncertain.

[CR43] Kaiser FG, Shimoda TA (1999). Responsibility as a predictor of ecological behaviour. J Environ Psychol.

[CR44] Kreuter MW, Green MC, Cappella JN (2007). Narrative communication in cancer prevention and control: a framework to guide research and application. Ann Behav Med.

[CR45] Leiserowitz A, Smith N, Marlon JR (2010). Americans’ knowledge of climate change.

[CR46] List JA, Shaikh AM, Xu Y (2019). Multiple hypothesis testing in experimental economics. Exp Econ.

[CR47] Lorenzoni I, Nicholson-Cole S, Whitmarsh L (2007). Barriers perceived to engaging with climate change among the UK public and their policy implications. Glob Environ Chang.

[CR48] Mallapaty S (2020) Animal source of the coronavirus continues to elude scientists. Nature. https://www.nature.com/d41586-020-01449-8. Accessed 12 June 2020 10.1038/d41586-020-01449-8 32427902

[CR49] Mayer RE (2002). Multimedia learning. Psychology of learning and motivation.

[CR50] Moezzi M, Janda KB, Rotmann S (2017). Using stories, narratives, and storytelling in energy and climate change research. Energy Res Soc Sci.

[CR51] Moyer-Gusé E (2008). Toward a theory of entertainment persuasion: explaining the persuasive effects of entertainment-education messages. Commun Theory.

[CR52] Pearson E, Tindle H, Ferguson M (2016). Can we tweet, post, and share our way to a more sustainable society? A review of the current contributions and future potential of #Socialmediaforsustainability. Annu Rev Env Resour.

[CR53] Pleasance C (2020) “All available evidence” suggests coronavirus came from animals: WHO. In: Mail Online. https://www.dailymail.co.uk/news/article-8240441/WHO-insists-available-evidence-suggests-coronavirus-came-animal.html. Accessed 12 June 2020

[CR54] Schwabish JA (2014). An economist’s guide to visualizing data. J Econ Perspect.

[CR55] Shen F, Sheer VC, Li R (2015). Impact of narratives on persuasion in health communication: a meta-analysis. J Advert.

[CR56] Shiller RJ (2017) Narrative economics. NBER Working Paper 23075, National Bureau of Economic Research

[CR57] Shreedhar G, Mourato S (2019). Experimental evidence on the impact of biodiversity conservation videos on charitable donations. Ecol Econ.

[CR58] Thompson A (2020) ‘We have disrespected animals and disrespected the environment’—Dame Jane Goodall on coronavirus. In: Channel 4 News. https://www.channel4.com/news/we-have-disrespected-animals-and-disrespected-the-environment-dame-jane-goodall-on-coronavirus. Accessed 12 June 2020

[CR59] van der Linden S (2015). The conspiracy-effect: exposure to conspiracy theories (about global warming) decreases pro-social behavior and science acceptance. Pers Individ Differ.

[CR60] van der Linden S, Maibach E, Leiserowitz A (2015). Improving public engagement with climate change: five “Best Practice” insights from psychological science. Perspect Psychol Sci.

[CR61] van Laer T, Feiereisen S, Visconti LM (2019). Storytelling in the digital era: a meta-analysis of relevant moderators of the narrative transportation effect. J Bus Res.

[CR62] Walker ME, Morera OF, Vining J, Orland B (1999). Disparate WTA–WTP disparities: the influence of human versus natural causes. J Behav Decis Mak.

[CR63] Winterbottom A, Bekker HL, Conner M, Mooney A (2008). Does narrative information bias individual’s decision making? A systematic review. Soc Sci Med.

